# Evaluation of Right Ventricular Function in Patients with Propionic Acidemia—A Cross-Sectional Study

**DOI:** 10.3390/children10010113

**Published:** 2023-01-05

**Authors:** Alexander Kovacevic, Sven F. Garbade, Friederike Hörster, Georg F. Hoffmann, Matthias Gorenflo, Derliz Mereles, Stefan Kölker, Christian Staufner

**Affiliations:** 1 Department of Pediatric and Congenital Cardiology, Heidelberg University Hospital, Im Neuenheimer Feld 430, 69120 Heidelberg, Germany; 2 Department of General Pediatrics, Division of Neuropediatrics and Metabolic Medicine, Heidelberg University Hospital, Im Neuenheimer Feld 430, 69120 Heidelberg, Germany; 3 Department of Cardiology, Angiology and Pulmology, Heidelberg University Hospital, Im Neuenheimer Feld 410, 69120 Heidelberg, Germany

**Keywords:** propionic acidemia, cardiac phenotype, right ventricular dysfunction, metabolic cardiomyopathy

## Abstract

(1) Background: In propionic acidemia (PA), myocardial involvement often leads to progressive cardiac dysfunction of the left ventricle (LV). Cardiomyopathy (CM) is an important contributor to mortality. Although known to be of prognostic value in CM, there are no published data on right ventricular (RV) function in PA patients. (2) Methods: In this cross-sectional single-center study, systolic and diastolic RV function of PA patients was assessed by echocardiography, including frequency, onset, and combinations of echocardiographic parameters, as well as correlations to LV size and function. (3) Results: *N* = 18 patients were enrolled. Tricuspid annulus S’ was abnormal in 16.7%, RV-longitudinal strain in 11.1%, tricuspid annular plane systolic excursion (TAPSE) in 11.1%, Tricuspid valve (TV) E/e’ in 33.3%, and TV E/A in 16.7%. The most prevalent combinations of pathological parameters were TV E/A + TV E/e’ and TAPSE + TV S’. With age, the probability of developing abnormal RV function increases according to age-dependent normative data. There is a significant correlation between TAPSE and mitral annular plane systolic excursion (MAPSE), and RV/LV-longitudinal strain (*p* ≤ 0.05). *N* = 5 individuals died 1.94 years (mean) after cardiac evaluation for this study, and all had abnormal RV functional parameters. (4) Conclusions: Signs of diastolic RV dysfunction can be found in up to one third of individuals, and systolic RV dysfunction in 16.7% of individuals in our cohort. RV function is impaired in PA patients with a poor outcome. RV functional parameters should be used to complement clinical and left ventricular echocardiographic findings.

## 1. Introduction

Propionic acidemia (PA), a rare organic aciduria inherited in an autosomal recessive pattern, is caused by deficiency of mitochondrial propionyl-CoA carboxylase. PA affects around 1/100,000–1/250,000 individuals in most regions. Clinical presentation and disease onset vary depending on residual enzymatic activity, intake of propiogenic precursors, and the occurrence of catabolism. Most PA patients have an early onset of disease, with ketoacidosis and hyperammonemia in the first days of life, typically presenting as poor feeding, decreased arousal, and progressive encephalopathy after a symptom-free period. Late onset patients present after the neonatal period. Both early and late onset patients often develop various organ manifestations of this multisystem disease, including global developmental delay and intellectual disability, muscular hypotonia, movement disorders, seizures, pancreatitis, and cardiac disease. While PA can be identified through expanded newborn screening, most infants are diagnosed during the neonatal period based upon clinical suspicion and biochemical workup. Diagnosis is confirmed via molecular genetic testing (PCCA and PCCB genes; PCC = propionyl-CoA carboxylase gene, subunits alpha and beta) [1,2,3,4,5,6,7,8].

Cardiac disease is among the most prevalent organ manifestations in PA and a major contributor to mortality. It presents mainly as progressive left ventricular (LV) dysfunction, often leading to manifest cardiomyopathy (CM), with life-threatening acute heart failure during, e.g., metabolic decompensation and/or infections. Further, acquired long QT syndrome is described with potential occurrence of malignant ventricular arrhythmias [1,2,3,4,5,6,7,8]. 

CM is found in 9–40% in PA, usually diagnosed by conventional echocardiography (by measuring LV fractional shortening). The treatment of cardiac disease consists of angiotensin converting enzyme inhibitors (ACE-I) and beta blockers, with anecdotal reports regarding Coenzyme Q10 [5,6,7,8]. 

In a recent study by our group, we found systolic and diastolic LV dysfunction in up to 72% of PA patients using advanced echocardiographic techniques [9].

Interestingly, there are no published data on right ventricular (RV) functional parameters in PA patients. This may reflect that during routine echocardiographic evaluation of PA patients, sonographers mainly focus on assessment of LV function [5,6,7,8]. However, RV systolic dysfunction is recognized as an independent predictor of the clinical outcome in patients with left ventricular systolic dysfunction due to other etiologies, such as ischemic heart disease or dilated CM (DCM) [10,11,12,13].

Due to the complex RV anatomy, measuring RV function is, however, more challenging compared to assessing LV function. Furthermore, LV contraction contributes to RV contraction [13,14]. Therefore, RV dysfunction may be also due to close interaction between LV and RV function and not attributed to predominant right heart failure, e.g., due to pulmonary hypertension or in right-sided obstructive lesions in congenital heart disease [13]. Both conditions are neither commonly seen in PA nor described as associated with PA [5,6,7,8,9].

To date, only data on LV functional parameters, mainly assessed by conventional echocardiography, have been published in PA. Therefore, we sought to investigate RV function in PA patients, including the impact of LV size and function on RV function, by advanced echocardiographic techniques.

## 2. Materials and Methods

This is a cross-sectional single-center study with a prospective observational design conducted in a Tertiary Medical Care Center. Ethical approval (S-525/2010) was provided by the local Ethical Board. Written informed consent for all patients was obtained. All individuals with confirmed PA (*n* = 18) were enrolled during the study period 2015–2020. The study individuals have been published before within a retrospective study on LV cardiac phenotype in PA [7], a prospective study on LV cardiac dysfunction in PA [9], and as part of larger studies on organic acidemias within the E-IMD project (please see Funding for further details) [3,15,16].

Transthoracic echocardiography was performed during routine outpatient visits according to current international guidelines by a single cardiologist following a predefined protocol [14,17,18]. Echocardiograms were made with Philips Diagnostic Ultrasound System; for details regarding software and sector array transducers, see [9].

Longitudinal systolic RV function was assessed by measuring tricuspid annular plane systolic excursion (TAPSE) by M-mode [19]. RV fractional area change (FAC) was calculated by tracing the endocardium in systole and diastole in an apical four-chamber view: FAC = 100% × (end-diastolic area − end-systolic area)/end-diastolic area [14,20]. S’ was calculated as the peak systolic velocity of the lateral tricuspid annular plane by pulsed Doppler tissue imaging from an apical four-chamber view [14,21]. RV myocardial performance index (RV-MPI) was assessed by pulsed Doppler imaging using the formula: RV MPI = TCO − ET/ET (ET right ventricular ejection time, TCO tricuspid valve closure to opening time) [14,21]. RV global longitudinal strain (RV-GLS; RV longitudinal strain estimated as average of seven segments including the interventricular septum in the pediatric age group) and RV free wall strain (RV-FWS; RV longitudinal strain estimated as average of three segments excluding the interventricular septum in the adult age group) was measured using two-dimensional speckle-tracking echocardiography (2D-STE) in a dedicated four-chamber view [14,22]. This approach for measuring RV longitudinal strain was chosen due to the availability of normal values for the different age groups, and in view of our available ultrasound and software equipment. GLS measurements were made as described before [9]. To evaluate RV diastolic function, we assessed Tricuspid valve (TV) inflow velocities by conventional Doppler and Tricuspid annulus early and late diastolic velocities by tissue Doppler (TDI): TV E/A, TV e’/a’ (the latter in adult patients only as not standardly used in children), and TV E/e’. RV-MPI is considered to represent both systolic and diastolic function, and its assessment is explained earlier [14,21]. Right ventricular end-diastolic diameters (RVEDD) were measured in a standard parasternal long axis view using motion-mode imaging (m-mode) in the pediatric age group. RV basal diameters were measured in a dedicated four-chamber view for the adult age group [14]. Z-scores for RV dimensions (for patients < 18 years) were calculated [23], and in addition compared to normal value ranges, according to Kampmann et al. [24]. Right atrial (RA) end-systolic dimensions were assessed by measuring RA area in the pediatric age group or RA minor axis and RA minor axis/body surface area (BSA) in the adult age group in a dedicated four-chamber view [25,26]. To compare RV and LV function, we looked at LV fractional shortening (FS) and ejection fraction by biplane Simpson’s, mitral annular plane systolic excursion (MAPSE), LV-GLS by STE, Mitral valve (MV) E/A ratio, MV E/e’, LV-MPI, and MV deceleration time (DT-E), as well as LV size. Parameters of RV and LV function were measured at the same examination and results of LV function were published earlier (for detailed description of methods and results, see reference [9]).

**Figure 1:**
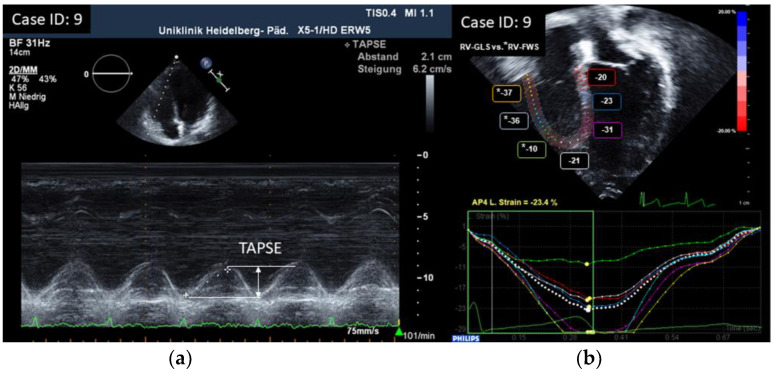
(**a**) Measurement of TAPSE by M-mode and (**b**) RV-GLS / RV-FWS* by 2D-STE.

**Figure 2:**
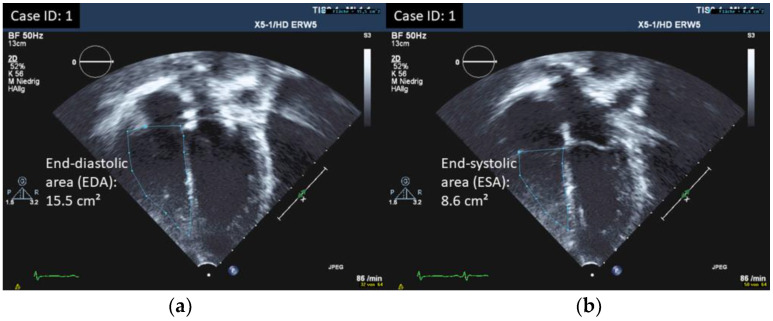
Measurement of RV-FAC (**a**) RV-EDA (**b**) ESA; FAC = 100% × (EDA − ESA)/EDA.

**Figure 3:**
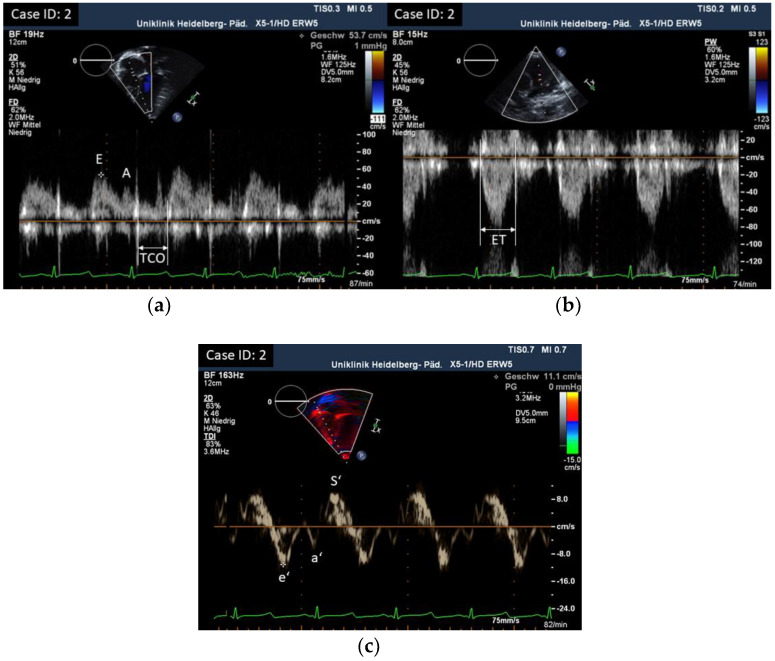
(**a**) TV E/A inflow and TCO and (**b**) Pulmonary valve (PV) Doppler and ET by conventional Doppler; RV-MPI = TCO − ET/ET. (**c**) Tissue Doppler imaging (TDI): TV e’, a’, and S’.

All results were compared to published age-dependent normative data [14,19,20,21,22,23,25,26]. Examples of echocardiographic measurements are displayed in Figure 1, Figure 2 and Figure 3.

To test for intra- and interobserver variability, randomly selected cases were reanalyzed and calculated by the equation: Variability % = (m1 − m2)/(m1 + m2)/2 × 100%, where m1 and m2 are the mean values of the first and the second measurement sets of one investigator (intra-observation variability) or the first measurement set between two investigators (inter-observation variability), who were blinded to each other’s results. The variables tested were LVEDD, MAPSE, TAPSE, MV E, MV e’, TV E, and TV e’ (Table S1).

### Statistics

Statistical analyses were computed with R language version 4.2.0. The linear relationship between two numeric variables was computed via Pearson correlation coefficient. Frequent combinations of echocardiographic parameters were displayed as combination of two to a maximum of four pathological parameters, according to the ECLAT algorithm implemented in R package ‘arules’. The development of specific pathological echocardiographic functional parameters of the RV were modeled as left and right censored data with the ‘Surv’-function in package ‘survival’: if a pathologic functional parameter were found, this observation was treated as left censored, otherwise as right censored.

## 3. Results

### 3.1. Study Cohort

Eighteen patients were enrolled. The median age at visit for cardiac evaluation was 13.1 years (range 0.6–28.1 years). The gender distribution was female *n* = 8 vs. male *n* = 10. Transthoracic echocardiography following a predefined protocol was performed during routine outpatient visits. None of the patients had structural heart disease (e.g., tricuspid/mitral valve disease or RV outflow tract obstruction). There were no cases of pulmonary hypertension. All patients had sinus rhythm during echocardiographic assessment, and none had acute preload changes. For further details regarding the study cohort, see reference [9].

### 3.2. Results of Echocardiographic Measurements

**Figure 4:**
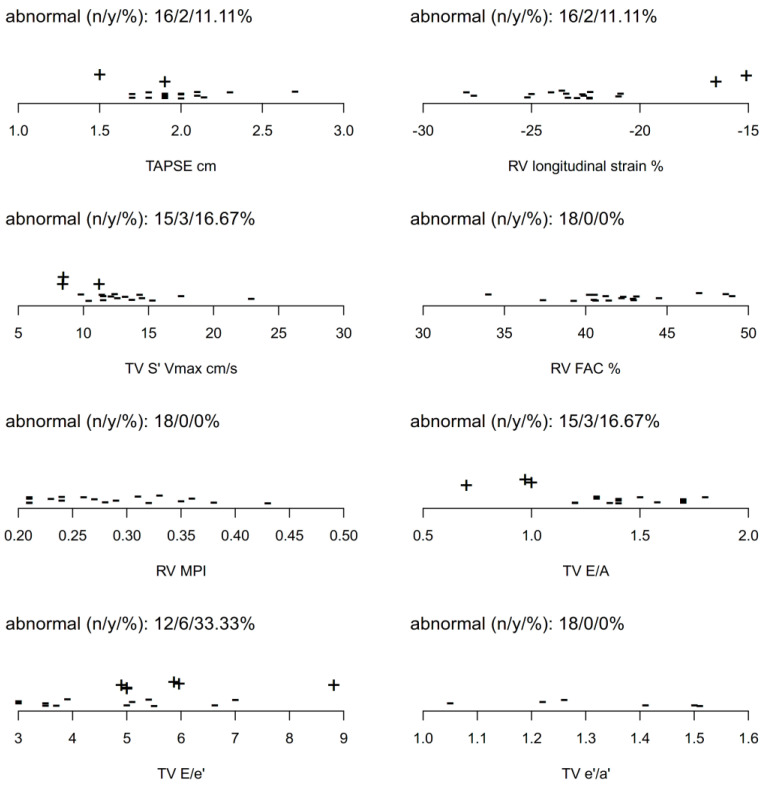
Distribution of raw data for TAPSE, RV longitudinal strain, TV S’, RV-FAC, RV MPI, TV E/A, TV E/e’, and TV e’/a’. “−“ = parameter within age-dependent reference range; “+” = parameter outside of age-dependent reference range (“abnormal”).

RV and RA size, diameter, and anatomy of the tricuspid valve annulus were normal in all included patients. Inspiratory collapse of the inferior vena cava was ≥50% in all cases. None of the presented cases had signs of elevated pulmonary artery pressure (none with a peak velocity of tricuspid valve regurgitation of more than 2.8 m/s). Tricuspid annulus S’ was abnormal in 16.7%, RV-strain in 11.1%, TAPSE in 11.1%, TV E/e’ in 33.3%, and TV E/A in 16.7%. Normal parameters were found in all individuals for RV-FAC, RV-MPI, and TV e’/a’ (TV e’/a’ assessed in adult patients). Table S2 and Table A1a,b summarize all echocardiographic parameters. In addition, Table 1 and Figure 4 give an overview of the distribution of the raw data. The most prevalent combinations of pathological parameters were TV E/A + TV E/e’ for diastolic RV function and TAPSE + TV S’ for systolic RV function; diastolic RV dysfunction was more prevalent (see Table A2 for data on support and count).

### 3.3. Effects of Patients’ Age on Right Ventricular Function

**Figure 5:**
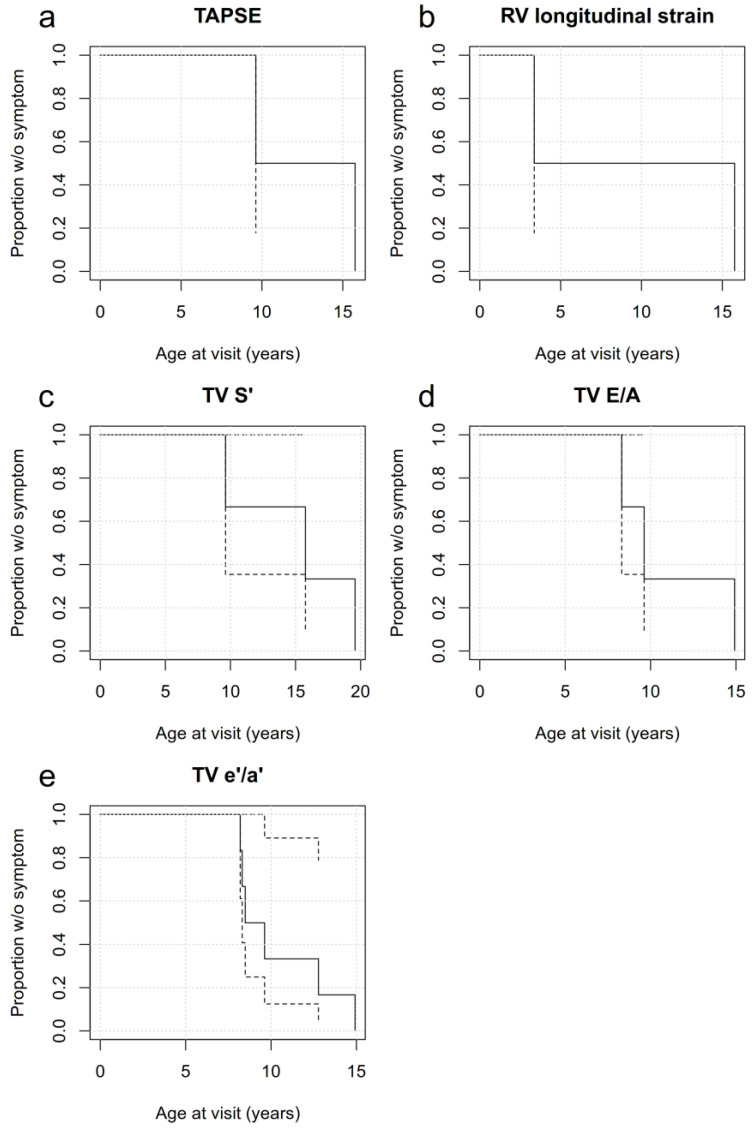
(**a**)–(**e**). Survival curves of proportions of patients without specific pathological RV functional parameters. With age, the probability of developing abnormal RV function increases according to age-dependent normative data. The dotted line indicates the 95% confidence interval.

With growing age, the proportion of patients without abnormal RV function decreases (Figure 5a–e).

### 3.4. Effect of Left Ventricular Size and Function on Right Ventricular Function

*N* = 3 individuals (ID 4, 13, 14) had a DCM with a LVEDD above the normal range and abnormal parameters for LV-FS, LV-EF, LV-MPI, and LV-GLS. Of these, *n* = 1 (ID 4) had additional abnormal RV-longitudinal strain, and another one (ID 14) had abnormal values for TAPSE, TV S’, and TV E/e’. The last one (ID 13) had no abnormal RV functional parameters. *N* = 1 individual (ID 9) had a dilated LV without pathological LV functional parameters; this patient also had normal values for all RV functional parameters. 

**Figure 6:**
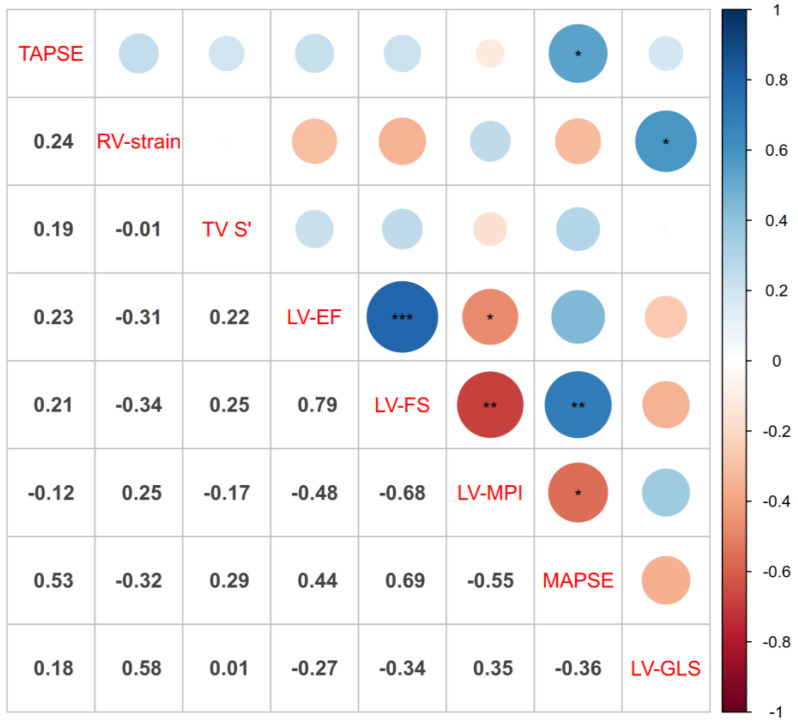
Correlation matrix: the size of the circles on the right side of the matrix correspond to more correlation, positive or negative (color code: blue positive and red negative correlation); the corresponding r values are displayed on the left side of the matrix. “*” corresponds to the calculated significance levels: * = *p* ≤ 0.05, ** = *p* ≤ 0.01, *** = *p* ≤ 0.001. Note that higher values of LV-MPI indicate cases with DCM, and that strain values are negative, i.e., values closer to zero indicate reduced, and more negative values better myocardial strain. For the remainder of displayed parameters, more positive values indicate better ventricular function.

Regarding the correlation of RV and LV function, there is a significant correlation between TAPSE and MAPSE, and RV longitudinal strain and LV-GLS (*p* ≤ 0.05). Correlation was more significant between LV functional parameters: LV-EF and LV-FS (*p* ≤ 0.001), LV-EF and LV-MPI (*p* ≤ 0.05), LV-FS and LV-MPI (*p* ≤ 0.01), LV-FS and MAPSE (*p* ≤ 0.01), and LV-MPI and MAPSE (*p* ≤ 0.05). Figure 6 shows the correlation matrix and Table S3 the corresponding correlation coefficients.

### 3.5. Patient Outcome and Right Ventricular Function

Until publication of this study, *n* = 5 patients (IDs 3, 4, 11, 14, 15) died (due to metabolic decompensation, pneumonia, other infection) with a mean time distance of 1.94 years (range 0.4–3.6 years) after cardiac evaluation for this study. Death occurred at a median age of 18 years (mean age 16.6 years, range 10–20 years).

Regarding RV functional parameters, ID 3 had pathological values for RV longitudinal strain, TAPSE, and TV S’. ID 4 had reduced RV longitudinal strain. ID 11 had abnormal TV E/A and TV E/e’. ID 14 had pathological TAPSE, TV S’, TV E/A, and TV E/e’. ID 15 had abnormal TV S’.

## 4. Discussion

In this study, we present novel data on systolic and diastolic RV function in PA patients. Up to one third of PA patients show signs of diastolic RV dysfunction, and 16% have systolic RV dysfunction, both in the absence of structural heart disease or pulmonary hypertension. With age, the probability of developing RV dysfunction, both diastolic and systolic, increases. Compared to our previous studies, RV dysfunction is less prevalent than LV dysfunction, which was found in up to 72% for both systolic and diastolic LV parameters in the same cohort.

RV dysfunction is frequently described, although less compared to LV dysfunction, e.g., in long-term adult survivors of childhood lymphoma and acute lymphoblastic leukemia [27], hypertensive heart failure [28], and also in β-thalassemia children [29], but prior to our present study not in PA patients. RV systolic dysfunction is recognized as independent predictor of clinical outcome e.g., in ischemic heart disease or DCM [10,11,12,13]. Some authors even state that the right ventricle and subsequent comprehensive evaluation of RV function seems “forgotten” in the routine work-up in pediatric CM [30]. The same authors found that RV systolic and diastolic functional parameters such as TAPSE, S′, e′/a′, and RV MPI were significantly correlated to LV-GLS [30]. This is supported by our findings of significant correlations between TAPSE and MAPSE, as well as RV and LV longitudinal strain.

Assessment of RV function should be considered important as it determines cardiac symptoms and exercise capacity in chronic heart failure, and it predicts the outcome in patients with DCM [10,11,12,13]. However, measuring RV function is more challenging due to the complex RV anatomy. The RV shares fibers in the interventricular septum with the LV. Subsequently, contraction of the interventricular septum augments RV contraction, described as systolic ventricular interaction. Therefore, RV dysfunction may also occur due to close interaction between LV and RV function [13]. In the absence of other causes of RV dysfunction, such as pulmonary hypertension or right-sided obstructive lesions, this concept, also known as ventricular interdependence, where the size and shape of one ventricle affects the function of the other ventricle, is likely the etiology of RV dysfunction we detected in our PA cohort. This is in line with the observed correlations between RV and LV dysfunction and—although less pronounced—the effects of LV size on RV function.

Cardiac disease is associated with high morbidity and mortality in PA patients. Further, disease progression may not be haltered by conventional therapies such as ACE-I or beta blockers started once CM is detected by conventional echocardiography [7], raising the need for earlier treatment and/or other new therapeutic strategies. Liver transplantation may reverse CM in PA, but can be associated with considerable mortality [6,31,32,33,34,35]. Predictors of outcome may help to guide patient management including decision for liver transplantation. In our cohort, five patients died till date. All had abnormal RV function at time of cardiac assessment, mainly impaired systolic RV function, whereas overall prevalence of RV dysfunction was 11–33%. It remains to be determined whether RV dysfunction can serve as an independent predictor of poor outcome in PA, in analogy to other conditions such as ischemic heart disease or DCM [10,11,12,13].

In summary, in view of our current and previous data [7,9] on cardiac phenotype in PA, we provide new insights regarding cardiac function in PA, the potential interaction between LV and RV function, and its possible effects on the outcome of this progressive disease. Considering the existing literature on cardiac function in PA, where LV function was mainly assessed by LV-FS [5,6,7,8], it seems important to define diagnostic methods such as advanced echocardiographic techniques to identify early and subtle signs of cardiac dysfunction and possibly parameters with predictive value. Due to limited case numbers per center for this rare disease, a multicenter approach would be desirable to confirm and expand the results from our single-center studies.

## 5. Limitations

With increasing age, PA patients are often noncooperative during echocardiography due to intellectual disability, and acoustic windows are hampered due to, e.g., immobility or scoliosis. We therefore (a) had to limit assessed echocardiographic parameters, and (b) quality of obtained views may have been impacted. However, intra- and interobserver variability for the echocardiographic parameters was excellent. There were no cardiac MRI or invasive data available, as not standardly recommended in PA. The strain algorithms used in QLAB have been validated for LV strain. However, algorithms used to determine strain are not chamber-specific, and, until 2018, both the apical four-chamber and the RV-focused four-chamber view were used to generate normal values for RV-GLS and FWS, and software packages designed for LV strain assessment were standardly used [36,37,38]. Additionally, it has been shown that RV strain measurements using LV-specific strain software correlate with RV strain measurements using RV-specific strain software [39]. Due to the study design, no longitudinal data were included. Therefore, effects of increasing age on RV function in individual study patients cannot be excluded.

## 6. Conclusions

We present, for the first time in literature, data on RV function in PA. We show that assessment of systolic and diastolic RV function is feasible, and that diastolic RV dysfunction occurs in up to one third of individuals in our cohort, whereas systolic RV dysfunction is found in 16.7%. RV dysfunction is less prevalent than LV dysfunction, and there is some correlation between LV and RV functional parameters. LV and subsequent RV dysfunction was found in PA patients with a poor outcome. In summary, RV functional parameters can be used to complement clinical and LV echocardiographic findings in the assessment of PA patients. A possible prognostic role of RV dysfunction in PA remains to be investigated.

## Figures and Tables

**Table 1:** Raw values of assessed RV functional parameters. Mean, median, 25% and 75% quantile, minimal (Min) and maximal (Max) values of right ventricular functional parameters in *n* = 18 individuals with PA. SD = standard deviation.

Parameter	*n*	Mean	SD	25%	Median	75%	Min	Max
TAPSE cm	18	1.96	0.26	1.83	1.90	2.08	1.50	2.70
TV S’ cm/s	18	12.84	3.41	11.25	12.25	14.15	8.40	22.90
RV-FAC %	18	42.09	3.68	40.51	41.80	43.07	34.00	49.00
RV-MPI	18	0.28	0.07	0.23	0.28	0.33	0.21	0.43
TV E/A	18	1.36	0.29	1.22	1.38	1.56	0.70	1.80
TV E/e’	18	5.04	1.51	3.75	5.00	5.78	3.00	8.82
RV strain %	18	−22.72	3.17	−23.98	−22.78	−22.31	−28.00	−15.10

## Data Availability

Further data will be made available on request.

## Supplementary Materials

The following supporting information can be downloaded at: https://www.mdpi.com/article/10.3390/children10010113/s1, Table S1: (a) Inter-observer variability of echocardiographic measurements; (b) Intra-observer variability of echocardiographic measurements, Table S2: Right ventricular and atrial size including Tricuspid valve annulus diameter of *n* = 18 individuals with PA, Table S3: Correlation coefficient r for the corresponding matrix of parameters (Figure 6).

## Appendix A

**Table A1:** (**a**)**.** Raw data of echocardiographic measurements for systolic RV function. (**b**)**.** Raw data of echocardiographic measurements for diastolic RV function.

**(a)**							
**ID**	**TAPSE cm**	**RV GLS % (ped.)**	**RV FWS % (adult)**		**TV S’ Vmax** **cm/s**	**RV FAC %**	**RV-MPI**
1	1.9	−22.6	/		12.6	44.5	0.24
2	1.7	−25	/		12.1	43.1	0.23
3	1.9	−16.5	/		11.2	41.4	0.32
4	2.7	−11.6	/		12.4	48.6	0.31
5	1.8	−24.1	/		11.4	40.5	0.21
6	1.8	/	−23.3		11.5	43.0	0.38
7	2	−23.4	/		11.5	41.2	0.21
8	1.9	−22.9	/		15.3	40.6	0.21
9	2.1	/	−27.7		14.5	42.2	0.29
10	1.9	−20.9	/		17.5	49.0	0.36
11	1.9	−21	/		22.9	42.9	0.35
12	2	/	−22.7		13.2	42.3	0.27
13	2	/	−22.3		10.4	39.3	0.43
14	1.5	−23.6	/		8.44	47.0	0.33
15	1.7	/	−22.3		8.4	37.4	0.21
16	2.3	/	−28.0		14.3	40.2	0.26
17	2.14	−25.2	/		13.7	40.5	0.28
18	2.1	−22.3	/		9.8	34.0	0.24
**(b)**							
**ID**	**TV E Vmax cm/s**	**TV A Vmax cm/s**	**TV E/A**	**TV e’ Vmax cm/s**	**TV a’ Vmax cm/s**	**TV e’/a’ (adult)**	**TV E/e’**
1	55	32	1.7	15.5	8	/	3.5
2	54	38	1.4	11	5.3	/	4.9
3	49	36	1.36	14	9.4	/	3.5
4	55	44	1.3	14	12	/	3.9
5	62	42	1.5	10.4	5.9	/	5.96
6	55	32	1.7	11	7.3	1.5	5
7	75	73	1	8.5	7	/	8.82
8	53	45	1.2	14.3	9.52	/	3.7
9	75	54	1.4	20.1	19	1.05	3
10	60	46	1.3	20	11	/	3
11	67.6	96.3	0.7	13.5	21.9	/	5
12	63	38	1.7	12.4	10.2	1.22	5.1
13	59	41.8	1.4	10.7	7.1	1.51	5.5
14	55.8	57.8	0.97	9.5	4.7	/	5.87
15	49.4	42.9	1.2	7.46	5.3	1.41	6.62
16	82	45	1.8	11.7	9.3	1.26	7
17	55	34.8	1.58	11	9.2	/	5
18	85	65.8	1.3	15.8	6.9	/	5.4

**Table A2:** First 10 itemsets (=combination of all pathological echocardiographic functional parameters) sorted by support and count. The most prevalent combination of pathological echocardiographic values was found for TV E/A + TV E/e’ and TAPSE + TV S’.

Items		Support	Count
1	{TV E/A, TV E/e’}	0.16666667	3
2	{TAPSE, TV S’}	0.11111111	2
3	{TAPSE, RV-strain, TV S’}	0.05555556	1
4	{RV-strain, TV S’}	0.05555556	1
5	{TAPSE, RV-strain}	0.05555556	1
6	{TAPSE, TV S’, TV E/A, TV E/e’}	0.05555556	1
7	{TAPSE, TV E/A, TV E/e’}	0.05555556	1
8	{TAPSE, TV S’, TV E/A}	0.05555556	1
9	{TAPSE, TV S’, TV E/e’}	0.05555556	1
10	{TAPSE, TV E/e’}	0.05555556	1
