# Supplementary material for: Evaluation of Right Ventricular Function in Patients with Propionic Acidemia—A Cross-Sectional Study
Source: Children (Basel). 2023 Jan 5;10(1):113. doi: 10.3390/children10010113 (PMC9856918; doi:10.3390/children10010113)
Supplement: Supplementary file 1 [file children-10-00113-s001.zip › Table S1.docx.pdf]

Table S1a. Interobserver variability

Case 1	LVEDD	MAPSE	MV E	MV e'	TAPSE	TV E	TV e'
AK1 - mean	3.3	1.2	98.5	13.6	2.1	55.0	11.0
MM - mean	3.1	1.3	97.0	13.4	2.1	54.8	10.1
Variability %	1.6	-1.2	0.4	0.4	0.5	0.1	2.1

Case 2	LVEDD	MAPSE	MV E	MV e'	TAPSE	TV E	TV e'
AK1 - mean	4.3	1.2	95.0	11.6	1.8	55.0	11.0
MM - mean	4.3	1.1	99.0	12.7	1.8	49.6	10.6
Variability %	-0.1	2.9	-1.0	-2.2	0.0	2.6	0.9

Case 3	LVEDD	MAPSE	MV E	MV e'	TAPSE	TV E	TV e'
AK1 - mean	4.2	1.1	121.0	11.0	1.7	54.0	11.0
MM - mean	4.0	1.1	122.3	9.9	1.9	55.3	12.1
Variability %	1.2	0.0	-0.3	2.6	-2.8	-0.6	-2.4

Case 4	LVEDD	MAPSE	MV E	MV e'	TAPSE	TV E	TV e'
AK1 - mean	4.1	1.1	78.0	9.1	1.9	60.0	20.0
MM - mean	3.9	1.1	82.5	11.3	2.2	67.9	19.4
Variability %	1.3	0.0	-1.4	-5.5	-3.3	-3.1	0.7

Table S1b. Intraobserver variability

Case 1	LVEDD	MAPSE	MV E	MV e'	TAPSE	TV E	TV e'
AK 1 - mean	3.3	1.2	98.5	13.6	2.1	55.0	11.0
AK 2 - mean	3.2	1.2	97.6	13.9	2.1	53.0	10.8
Variability %	0.8	0.2	0.2	-0.6	0.1	0.9	0.5

Case 2	LVEDD	MAPSE	MV E	MV e'	TAPSE	TV E	TV e'
AK 1 - mean	4.3	1.2	95.0	11.6	1.8	55.0	11.0
AK 2 - mean	4.3	1.2	97.0	12.2	1.7	54.0	10.0
Variability %	-0.3	0.0	-0.5	-1.3	1.4	0.5	2.4

Case 3	LVEDD	MAPSE	MV E	MV e'	TAPSE	TV E	TV e'
AK 1 - mean	4.2	1.1	121.0	11.0	1.7	54.0	11.0
mean	4.1	1.1	118.7	10.7	1.9	56.2	11.8
Variability %	0.6	0.0	0.4	0.7	-2.8	-0.9	-1.8

Case 4	LVEDD	MAPSE	MV E	MV e'	TAPSE	TV E	TV e'
AK 1 - mean	4.1	1.1	78.0	9.1	1.9	60.0	20.0
mean	4.1	1.2	77.5	9.3	1.9	62.3	20.0
Variability %	-0.2	-2.2	0.2	-0.5	0.0	-0.9	0.0
