# Supplementary material for: Evaluation of Right Ventricular Function in Patients with Propionic Acidemia—A Cross-Sectional Study
Source: Children (Basel). 2023 Jan 5;10(1):113. doi: 10.3390/children10010113 (PMC9856918; doi:10.3390/children10010113)
Supplement: Supplementary file 1 [file children-10-00113-s001.zip › Table S2.docx.pdf]

Table S2. Right ventricular and atrial size including Tricuspid valve annulus diameter of n=18 individuals with PA. Z-scores, RVEDD according to Kampmann et al., RA area for pediatric patients, the remainder for adults; this approach was chosen due to the availability of normal values; n=normal.

ID	RVEDD cm (ped.)	RV z-score (ped.)	RVEDD according to Kampmann et al. (ped.)	RV basal diameter, mm (adult)	TV annulus cm	TV z-score (ped.)	RA area cm² (ped.)	RA minor axis cm (adult)	RA minor axis/BSA cm/m² (adult)
**1**	1.2	-1.8	n	/	2.4	-0.44	7.9	/	/
**2**	1.2	-1.5	n	/	2.3	-0.65	7.7	/	/
**3**	1.4	-1.9	n	/	3.4	0.51	14.1	/	/
**4**	1.3	-1.9	n	/	2.8	-0.14	11.7	/	/
**5**	1.5	-0.68	n	/	2.2	-0.76	8.7	/	/
**6**	/	/	/	26	2.4	/	/	3	2
**7**	1.2	-1.7	n	/	2.2	-1	7.3	/	/
**8**	1.7	-0.64	n	/	2.4	-0.85	10.9	/	/
**9**	/	/	/	34	3.2	/	/	3.5	2.06
**10**	1.3	-1.6	n	/	2.4	-0.68	9	/	/
**11**	1.3	-1.7	n	/	2.8	-0.01	12.5	/	/
**12**	/	/	/	27	2.5	/	/	2.9	2.07
**13**	/	/	/	30	3	/	/	4	2.22
**14**	1.2	-1.7	n	/	2.3	-0.75	9.6	/	/
**15**	/	/	/	34	2.5	/	/	3.3	2.36
**16**	/	/	/	26	3.1	/	/	3.3	1.94
**17**	1	-1.51	n	/	2.1	0.17	6.5	/	/
**18**	1.1	-0.64	n	/	1.7	0.08	4.8	/	/
