# Supplementary material for: Evaluation of Right Ventricular Function in Patients with Propionic Acidemia—A Cross-Sectional Study
Source: Children (Basel). 2023 Jan 5;10(1):113. doi: 10.3390/children10010113 (PMC9856918; doi:10.3390/children10010113)
Supplement: Supplementary file 1 [file children-10-00113-s001.zip › Table S3.docx.pdf]

Table S3: Correlation coefficient r for the corresponding matrix of parameters (Figure 6).

**Parameters**	**TAPSE**	**RV-strain**	**TV S'**	**LV-EF**	**LV-FS**	**LV-MPI**	**MAPSE**	**LV-GLS**
**TAPSE**	1	0.24	0.19	0.23	0.21	-0.12	0.53	0.18
**RV-strain**	0.24	1	-0.01	-0.31	-0.34	0.25	-0.32	0.58
**TV S'**	0.19	-0.01	1	0.22	0.25	-0.17	0.29	0.01
**LV-EF**	0.23	-0.31	0.22	1	0.79	-0.48	0.44	-0.27
**LV-FS**	0.21	-0.34	0.25	0.79	1	-0.68	0.69	-0.34
**LV-MPI**	-0.12	0.25	-0.17	-0.48	-0.68	1	-0.55	0.35
**MAPSE**	0.53	-0.32	0.29	0.44	0.69	-0.55	1	-0.36
**LV-GLS**	0.18	0.58	0.01	-0.27	-0.34	0.35	-0.36	1
